# The non-coding variant rs1800734 enhances DCLK3 expression through long-range interaction and promotes colorectal cancer progression

**DOI:** 10.1038/ncomms14418

**Published:** 2017-02-14

**Authors:** Ning Qing Liu, Menno ter Huurne, Luan N. Nguyen, Tianran Peng, Shuang-Yin Wang, James B. Studd, Onkar Joshi, Halit Ongen, Jesper B Bramsen, Jian Yan, Claus L. Andersen, Jussi Taipale, Emmanouil T. Dermitzakis, Richard S. Houlston, Nina C. Hubner, Hendrik G. Stunnenberg

**Affiliations:** 1 Faculty of Science, Department of Molecular Biology, Radboud University, RIMLS, PO BOX 9101, 6500HB Nijmegen, The Netherlands; 2 Division of Genetics and Epidemiology, Institute of Cancer Research, 15 Cotswold Road, Sutton, SM2 5NG Surrey, UK; 3 Department of Genetic Medicine and Development, University of Geneva Medical School, Geneva 1211, Switzerland; 4 Department of Molecular Medicine, Aarhus University Hospital, Palle Juul-Jensens Boulevard 99, DK-8200 Aarhus, Denmark; 5 Division of Functional Genomics and Systems Biology, Department of Medical Biochemistry and Biophysics, Karolinska Institutet, SE 141 83 Stockholm, Sweden; 6 Ludwig Institute for Cancer Research, 9500 Gilman Drive, La Jolla, California 92093, USA

## Abstract

Genome-wide association studies have identified a great number of non-coding risk variants for colorectal cancer (CRC). To date, the majority of these variants have not been functionally studied. Identification of allele-specific transcription factor (TF) binding is of great importance to understand regulatory consequences of such variants. A recently developed proteome-wide analysis of disease-associated SNPs (PWAS) enables identification of TF-DNA interactions in an unbiased manner. Here we perform a large-scale PWAS study to comprehensively characterize TF-binding landscape that is associated with CRC, which identifies 731 allele-specific TF binding at 116 CRC risk loci. This screen identifies the A-allele of rs1800734 within the promoter region of *MLH1* as perturbing the binding of TFAP4 and consequently increasing *DCLK3* expression through a long-range interaction, which promotes cancer malignancy through enhancing expression of the genes related to epithelial-to-mesenchymal transition.

## Abstract (web summary)

Functional characterisation of non-coding risk variants for colorectal cancer is missing. Here the authors conduct a large-scale proteome-wide analysis of disease associated SNPs to characterize allele-specific transcription factor binding landscape in colorectal cancer and identify one functionally significant SNP.

An individual's risk to develop colorectal cancer (CRC) is affected by a broad spectrum of genetic variants that abolish the functions and/or alter the expression of target genes. In CRC, two types of genetic variants have been extensively discussed to contribute to disease onset and progression: (1) protein-coding mutations and (2) non-coding variants, in particular in DNA regulatory elements. To date, the majority of studies have focused on protein-coding mutations. Key coding mutations such as *APC/CTNNB1, KRAS/BRAF, PIK3CA, TP53* and *SMAD4* have been intensively characterized^1^. However, even though a great number of non-coding risk variants for CRC have been identified in genome-wide association studies (GWAS)^2^,^3^,^4^,^5^,^6^,^7^,^8^,^9^, their molecular functions have rarely been determined.

Functional genetic variants in distal DNA regulatory elements may alter transcription networks in several ways such as by affecting transcription factor (TF) binding. Single-nucleotide polymorphisms (SNPs) within TF-binding sites may affect the local chromatin accessibility^10^,^11^,^12^ and/or alter the expression of gene targets through mediating different chromatin interactions^13^,^14^,^15^. Therefore, identification of variant-specific TF interactors is of great importance to understand regulatory consequences of the variants. However, DNA oriented methods such as DNase I sequencing (DNase I-seq)^16^, systematic evolution of ligands by exponential enrichment sequencing^17^ and chromatin immunoprecipitation with massively parallel DNA sequencing (ChIP-seq)^18^ are biased by DNA motif knowledge or limited by the availability of antibodies, resulting in a biased identification of TF-binding dynamics at disease-associated loci. A recently developed proteome-wide analysis of disease-associated SNPs (PWAS) enables to identify DNA–TF interactions in an unbiased manner^19^. A similar approach has been used to characterize a protein–DNA interaction map for ultra-conserved elements^20^. Therefore, we performed a large-scale PWAS study to comprehensively understand TF-binding landscape related to CRC.

As the outcome from our PWAS screen, we further investigated the functions of a SNP located in the promoter region of *MLH1* gene (*MLH1*-93G>A or rs1800734), which has been associated with the risk of several cancer types including CRC^9^,^21^,^22^, endometrial cancer^23^, glioblastoma^24^ and lung cancer^25^. Here we identified a molecular function of this SNP in promoting cancer malignancy through a novel gene target named *DCLK3*.

## Results

### PWAS identified TF occupancy switching at the 116 loci

**Figure 1:**
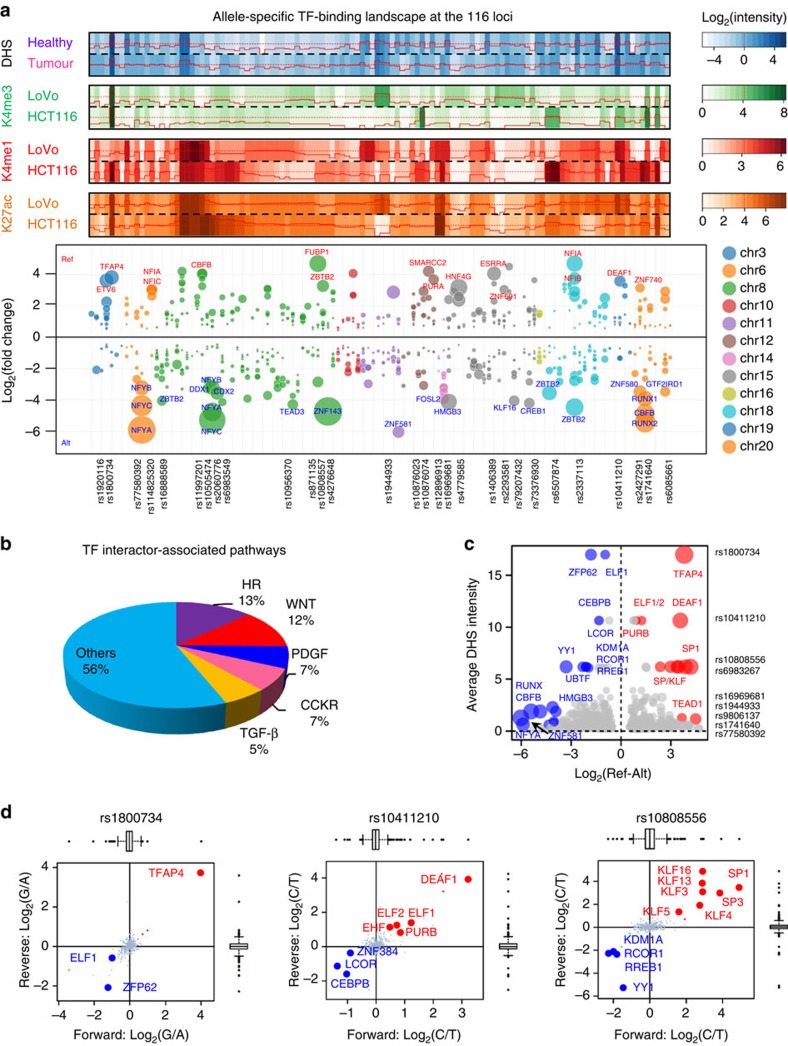
PWAS screen systematically identified allele-specific TF binding at the selected CRC risk loci. (**a**) Allele-specific binding of the 731 candidate TFs at the 116 CRC risk loci. Chromatin environment of the 116 SNPs was described by DHS of these loci in the 15 fetal large intestine tissues and 12 CRC cell lines, and histone modifications (H3K4me3, H3K4me1 and H3K27ac) within ±1 kb regions around these loci in the LoVo and HCT116 cell lines. The TFs with *P*-value<10^−30^ and absolute Log_2_(fold change)>3 was listed in the bubble plot. Bubble size represents −Log_10_(*P*-values) of the interactors in the pull-down screen (*n*=2 pulldowns per SNP, *P*-values: A/B significance test). (**b**) Pathway annotation of the 731 TFs (HR, gonadotropin-releasing hormone receptor pathway; WNT/PDGF/CCKR/transforming growth factor (TGF)-β, Wnt/PDGF/Cholecystokinin/TGF-β signalling pathways). (**c**) TF–SNP interactions ranked by fold changes in the PWAS screen and DHS at the SNP loci. Bubble size indicates the −Log_10_(*P*-values) of the TF–SNP interactions (*n*=2 pulldowns per SNP, red and blue bubbles: *P*-value<0.05, *Z*-test). (**d**) The top three candidate TF–SNP interactions (*n*=2 pulldowns per SNP, red and blue dots: *P*-values<0.01, A/B significance test).

We selected 116 SNPs associated with CRC risk^2^,^3^,^4^,^5^,^6^,^7^,^8^,^9^ for PWAS analysis including the following: (1) typed and imputed GWAS significant SNPs (for imputed SNPs, linkage disequilibrium (LD) *r*^2^≥0.2) from 8q24.21 (MYC-335), 15q13.3 (*GREM1*) and 18q21.1 (*SMAD7*); (2) SNPs with functional evidence rs16969681/15q13.3 (ref. 26), rs58920878/18q21.1 (ref. 27), rs16888589/8q23.3 (ref. 28) and rs4444235/14q22.2 (ref. 29); (3) 3 SNPs from 3p22.2 (*MLH1* region)^9^; and (4) GWAS significant SNPs in DNase I-seq peaks in minimal one of 15 fetal large intestine tissues and 12 CRC cell lines (Supplementary Data 1). The PWAS analysis identified 731 TF-binding alterations between reference (Ref) and alternate (Alt) alleles (*P*<0.01, A/B significance test) (Supplementary Data 2). Compared with proteome data, TF–DNA interactome data showed a clear enrichment for TFs (Supplementary Fig. 1a) and the altered binding events mediated by known TFs (Supplementary Data 3) showed stronger allele preference than other interactions (*P*=4.4 × 10^−6^, Mann–Whitney *U*-test) (Supplementary Fig. 1b). Overlay of the pulldowns showed a consistent allele preference between two replicate experiments (Supplementary Fig. 1c). Many of the 731 TFs showed >8-fold affinity to one of the alleles at these loci (Fig. 1a), for example, TFAP4 at rs1800734 and RUNX1/RUNX2/CBFB at rs1741640. As expected, top pathways associated with the 731 TFs included key CRC drivers such as WNT and transforming growth factor-β pathways (Fig. 1b).

It is well-known that DNase I-hypersensitive sites coincide with regulatory elements and are the hotspots for TF binding^16^. To better predict *in vivo* TF binding, it is necessary to consider the hypersensitivity of tested regions. Therefore, we ranked all the SNP-TF interactions based on PWAS fold change (Ref/Alt allele) and DNase I hypersensitivity (DHS) of the SNP loci. A total of 27 significant allele-specific SNP–TF binding events were considered to be important (Fig. 1c and Supplementary Table 1). Many selected TF–SNP interactions (Fig. 1d and Supplementary Fig. 1d) were validated using ChIP-seq data (Supplementary Fig. 1e). Based on this selection, the top candidate SNP is rs1800734 (*MLH1* -93 G>A) in the 5′-untranslated region (UTR) region of *MLH1* gene. Therefore, we decided to focus on this SNP.

### PWAS identified specific interactors of rs1800734

**Figure 2:**
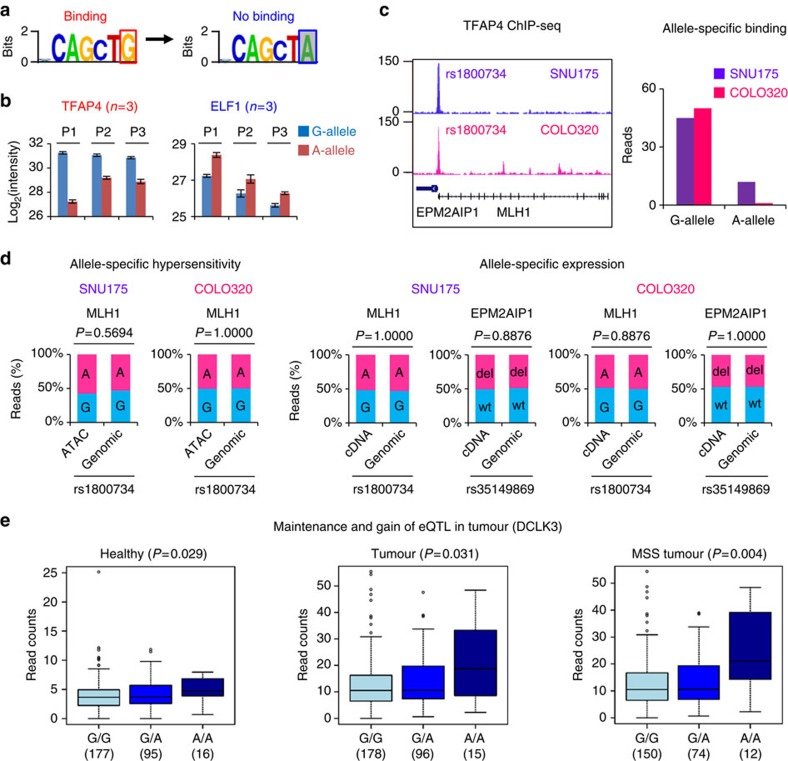
Identification of allele-specific interactors for rs1800734 and its potential gene targets. (**a**) Motif analysis interpreted that A-allele of the loci perturbs E-box motif. (**b**) The specific binding of TFAP4 (P1=2.0 × 10^−6^, P2=3.1 × 10^−5^, P3=7.8 × 10^−5^, Student's *t*-test) and ELF1 (P1=2.1 × 10^−4^, P2=0.011, P3=6.9 × 10^−4^, Student's *t*-test) on G- and A-allele was observed in the three different cell lines (data are represented as mean and error bars indicate s.d., *n*=3 pulldowns per SNP). ZFP62 binding on A-allele, as shown in Fig. 1d, was not consistent in all the three cell lines and was hence not considered as the general TF regulator at this locus. (**c**) This allele-specific binding was validated by ChIP-seq of TFAP4. This SNP did not change (**d**) the local chromatin accessibility and the expression of two *cis*-regulated genes (*MLH1* and *EPM2AIP1*) (G (G-allele) and A (A-allele) of rs1800734, wt (wild-type allele) and del (deletion allele) of rs35149869, *P*-values: Fisher's exact test). (**e**) eQTL analysis revealed a novel gene targets *DCLK3* of this SNP (*P*-values were calculated in FastQTL software based on linear regression model).

Our PWAS screen identified TFAP4 as an allele-specific interactor with an almost 16-fold higher affinity for G-allele, whereas ELF1 showed higher affinity for the A-allele of rs1800734 in LoVo cells (Fig. 1d). The A-allele abolishes TFAP4 binding due to a point mutation at the last position of the E-box (Fig. 2a), which simultaneously creates an E26 transformation-specific (ETS) family binding motif (Supplementary Fig. 2a). We corroborated and extended this finding by label-free-based DNA pulldown with SNU175 and COLO320 extracts (Fig. 2b and supplementary Fig. 2b). Notably, other basic helix-loop-helix (bHLH) and ETS family members also displayed allele-specific binding, including MYC and ELF2 (Supplementary Fig. 2b,c), indicating that these TFs can compete with TFAP4 and ELF1 at this locus. Using ChIP-seq as an orthogonal technique, we validated TFAP4 and ELF1 binding at the rs1800734 in the SNU175 and COLO320, cell lines heterozygous for this locus. Consistent with PWAS, TFAP4 ChIP-seq showed a dominant preference for G-allele binding in the two cell lines (Fig. 2c). ELF1 did not show significant allele-binding preference (Supplementary Fig. 2d), which may be due to the competitive binding interference by other ETS proteins.

### *DCLK3* is a novel target of rs1800734

Given the position of the SNP in the promoter of *MLH1*, we further investigated whether predisposition of rs1800734 in CRC is due to DNA methylation of *MLH1* promoter as proposed^9^,^30^. We tested hypersensitivity and transcription of G- and A-allele in the two heterozygous cell lines, which showed the two alleles are equally accessible and transcribed (Fig. 2d). The neighbouring gene *EPM2AIP1*, also reported to be regulated by rs1800734 (ref. 31), was similarly unaffected by rs1800734 (Fig. 2d). Hence, we conclude that rs1800734 does not result in allele-specific epigenetic silencing of either *MLH1* or *EPM2AIP1* in these cell lines. We sought to confirm our observation in the Systems Biology of Colorectal Cancer (SYSCOL) cohort of paired healthy and tumour tissues (healthy: *n*=288, tumour: =289). A strong expression quantitative trait loci (eQTL) between rs1800734 and MLH1 was observed in the healthy tissues (*P*=0.001, linear regression model) but the A-allele was associated with increased *MLH1* expression. This eQTL was lost in the tumours and remained only a weak association in microsatellite stable (MSS) tumours (*P*=0.025, *n*=236, linear regression model) (Supplementary Fig. 3a). Intriguingly, we identified a correlation between rs1800734 and DCLK3 expression in the healthy (*P*=0.029, linear regression model) and tumour (*P*=0.031, linear regression model) samples, and this association was highly significant in the MSS patients (*P*=0.004, linear regression model) (Fig. 2e), indicating this locus may act as a distal enhancer and regulate *DCLK3*.

### The A-allele positively regulates transcription of *DCLK3*

**Figure 3:**
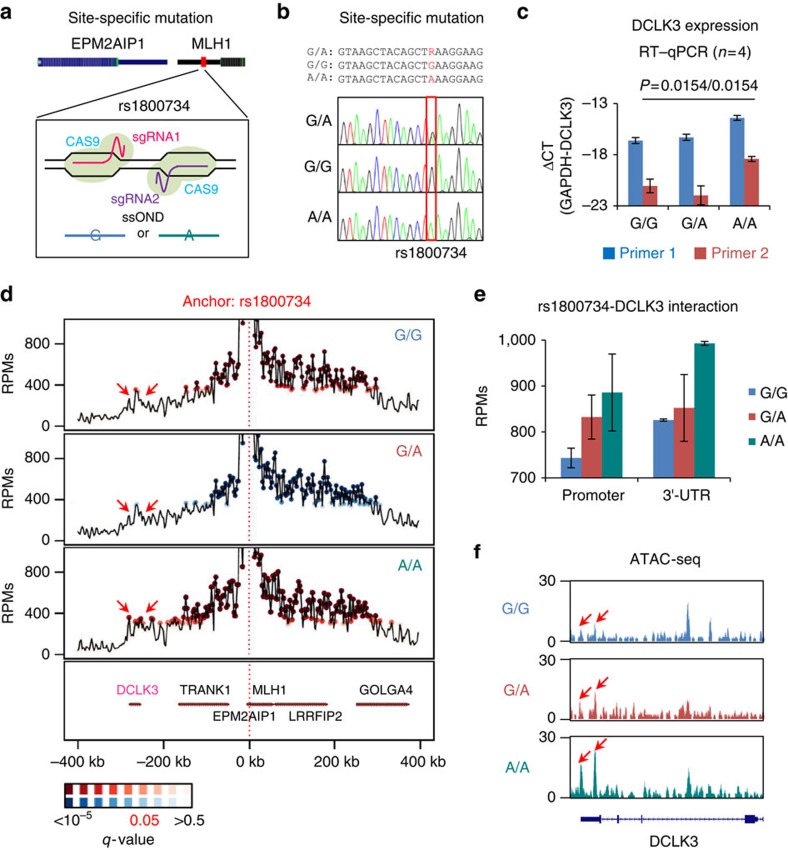
Molecular regulatory mechanisms of rs1800734 on *DCLK3*. (**a**) Isogenic cell lines possessing homozygous mutations (G/G and A/A) at rs1800734 were generated and (**b**) confirmed, and (**c**) the expression of *DCLK3* was elevated in the A/A cell line (data are represented as mean and error bars indicate s.d., *n*=4 biological replicates per cell line, *P*-values: Student's *t*-test). (**d**) 4C-seq identified chromatin interactions between rs1800734 and the *DCLK3* region. (**e**) The A/A cell line showed stronger chromatin interaction than other cell lines (data are represented as mean and error bars indicate s.d., *n*=2 biological replicates per cell line). (**f**) The enhanced chromatin interaction results in increased chromatin accessibility at 3′-UTR region of *DCLK3* in the A/A line.

To establish the functional relation between rs1800734 and *DCLK3* expression, two isogenic cell lines homozygous for G- or A-allele were generated from COLO320 using CRISPR-CAS9 technique (Fig. 3a). Successful targeting of rs1800734 was confirmed by Sanger sequencing (Fig. 3b). No other mutation was observed in the surrounding region. ChIP–quantitative PCR (qPCR) confirmed higher TFAP4 binding to the G-allele (Supplementary Fig. 3b) and the level of *MLH1* and *EPM2AIP1* transcription was identical in the three isogenic lines (Supplementary Fig. 3c). Importantly, the eQTL association between rs1800734 and *DCLK3* expression was replicated and a fivefold difference in *DCLK3* expression was observed between G- and A-homozygotes (Fig. 3c). By sequencing the inter-exonic reverse transcriptase–qPCR products of DCLK3, we confirmed the transcription of *DCLK3* in these cell lines (Supplementary Fig. 3d).

### rs1800734 regulates DCLK3 through long-range interactions

A capture Hi-C study has suggested that rs3806624 in the promoter of *EOMES* affects *AZI2*, a gene 640 kbp downstream to this SNP, through a long-range chromatin interaction^14^. A similar constellation may apply to rs1800734. Therefore, Circularized Chromosome Conformation Capture with massively parallel DNA sequencing (4C-seq) was employed to search for long-range interactions between rs1800734 and other potential targets in the three isogenic cell lines. Using rs1800734 as a view point, a significantly enhanced interaction was observed in A-homozygote with *DCLK3* region (Fig. 3d). Increased interactions were found in the promoter and 3′-UTR region of DCLK3 in two independent experiments (Fig. 3e) and, in addition, the 3′-UTR interaction appeared to increase chromatin accessibility (Fig. 3f). In conclusion, the A-allele of rs1800734 increases the *DCLK3* transcription through increased chromatin interaction and enhanced chromatin accessibility.

### DCLK3 is a potential oncogenic and tumour progressive factor

**Figure 4:**
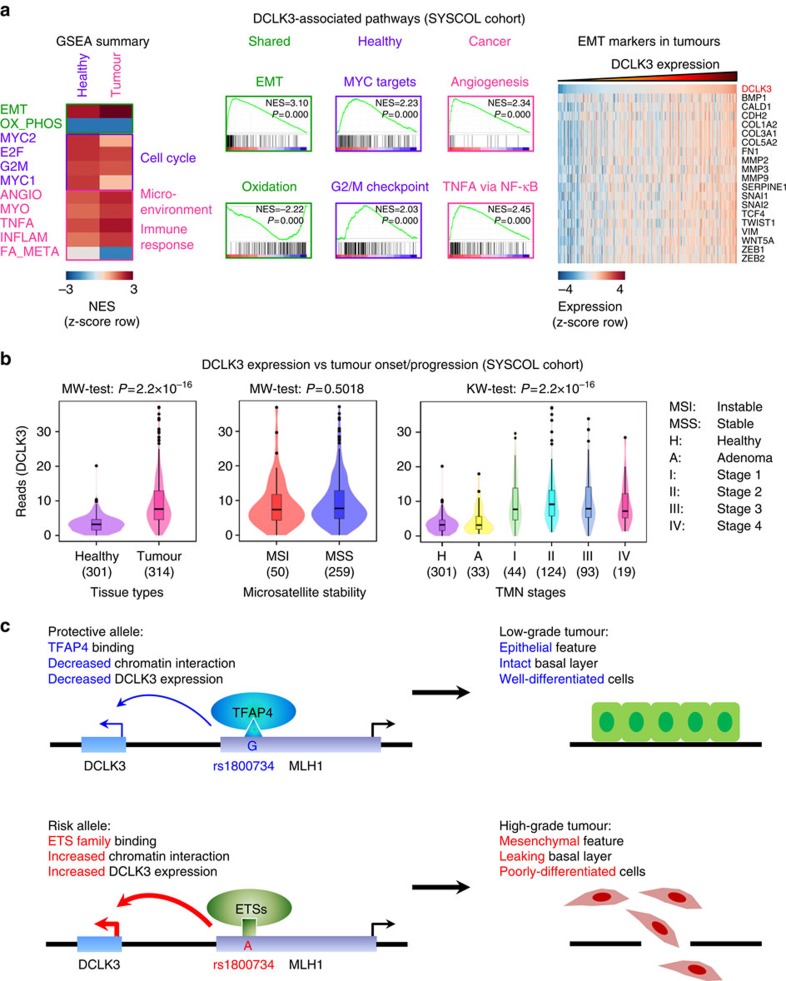
Identification of DCLK3-associated gene sets and clinical parameters in the SYSCOL RNA-seq cohort. (**a**) GSEA analysis revealed DCLK3 correlated cancer hallmark gene sets. EMT and oxidative phosphorylation-associated gene sets were highly significantly associated with the *DCLK3* expression in both healthy and tumour tissues. In addition, some DCLK3-associated gene sets showed healthy or tumour tissue specificity (*P*-values were calculated in GSEA based on Pearson's correlation). The expression of key EMT markers showed good correlation with the *DCLK3* expression. (**b**) The *DCLK3* expression was preferentially elevated in the tumours and especially the malignant tissues. However, we did not observe a clear difference between MSI and MSS tumours (*P*-values: Mann–whitney *U*-test (MW test) and Kruskal–Wallis test (KW test)). (**c**) A proposed model describing how rs1800734 modifies the risk of CRC malignancy. TFAP4 and ETS family members specifically bind to the protective G- or risk A-allele, respectively. The rs1800734-ETS interaction increases the enhancer activity of the rs1800734 locus and enhances the expression of *DCLK3* through an increased chromatin interaction. Cancer cells with the elevated *DCLK3* expression undergo EMT and therefore metastasize to distal sites.

DCLK3 is one of three doublecortin-like kinases (DCLK1, DCLK2 and DCLK3). In this family, DCLK1 has been shown to be a cancer stem cell marker in intestinal tumours^32^. The molecular function of the DCLK3 has not been characterized in depth. We therefore performed gene set enrichment analysis (GSEA) to identify DCLK3-associated gene sets in the SYSCOL RNA sequencing (RNA-seq) cohort. Interestingly, we found that epithelial-to-mesenchymal transition (EMT)-related genes were highly correlated to the expression of *DCLK3* in the healthy tissues (normalized enrichment score (NES)=2.50), which was enhanced in the tumour samples (NES=3.10) (Fig. 4a and Supplementary Table 2). Using a cutoff of NES at 2.00, we also identified gene sets that are preferentially enriched in the healthy versus tumour tissues (Supplementary Table 2), for example ‘MYC targets' and ‘G2/M checkpoint' gene sets were enriched in the healthy tissue, whereas ‘Angiogenensis' and ‘tumor necrosis factor alpha (TNFA) signalling via nuclear factor–κB' gene sets were enriched in the tumour tissues (Fig. 4a). As expected, common EMT markers such as CALD1, FN1, SNAI1, SNAI2, TWIST1, VIM, ZEB1 and ZEB2 were highly significantly co-expressed with DCLK3 in the tumours (Fig. 4a), indicating that DCLK3 is an EMT regulator. Furthermore, we observed elevated *DCLK3* expression in the tumour compared with healthy tissues (*P*=2.2 × 10^−16^, Mann–Whitney *U*-test) (Fig. 4b), but no difference between the microsatellite instable (MSI) and microsatellite stable (MSS) tumours (Fig. 4b). Interestingly, this elevation appeared to be correlated with the CRC progression: *DCLK3* expression was at a comparable level in the healthy and adenoma tissues, and increased ∼2-fold in the stage I tumours and remained at this level during tumour malignancy (*P*=2.2 × 10^−16^, Kruskal–Wallis test) (Fig. 4b). Therefore, DCLK3 may promote EMT events and consequently drive tumour malignancy.

## Discussion

Our study generated the first TF–SNP interaction map at presumed disease-relevant loci of CRC and determined TF-binding occupancy at the 116 upmost relevant CRC risk loci. Together with GWAS and epigenetic profiling data, our PWAS screens provide a comprehensive TF-binding landscape of these loci and yielded candidate interactions for further functional investigations (Fig. 1a,c,d and Supplementary Fig. 1d,e). As an alternative tool to investigate allele-specific TF-binding events, the PWAS approach shows several advantages over the DNA-centric methods. In contrast to ChIP-seq technique, PWAS-based identification is a hypothesis-free approach, which does not require any knowledge on the possible binding TFs at risk loci. In addition, application of PWAS approach is not limited by the availability of high-quality ChIP-grade antibodies. Even though large ChIP-seq data sets have been generated^18^,^33^, these data nevertheless cover only a small proportion of the entire repertoire of TFs. Other DNA-centric methods, such as DNase I-seq or assay for transposase-accessible chromatin with high throughput sequencing (ATAC-seq), are only capable of identifying DNA motifs present at risk loci, which makes it difficult to predict actual binding TF(s) from a family sharing similar motifs in a specific cell type. The outcome of PWAS is a reflection of TF abundance, TF ability to bind to the sequence (affinity), as well as synergistic and antagonist effects due to binding of other TFs to adjacent or overlapping sequences. Therefore, actual binding TFs in a given cell type can be predicted using PWAS method. In addition, PWAS approach also help to identify binding TFs at the loci only partially matching consensus motif sequences and therefore cannot be predicted by motif prediction-based methods.

Based on the PWAS screen, we investigated in great depths for a well-known CRC-associated SNP: *MLH1*-93G>A or rs1800734. It has been postulated that A-allele (risk allele) of the rs1800734 recruits repressive TFs, which subsequently results in promoter methylation of the *MLH1* gene^9^,^30^, supported by an association between the A-allele of the rs1800734 and promoter methylation^34^ or decreased expression of *MLH1* (refs 35, 36). However, Suter *et al*.^37^ showed contradictory results that A-allele of this SNP is associated with lower promoter methylation and higher transcription of *MLH1*. Our results suggested that TFAP4 preferentially binds to T-allele of the rs1800734 but does not change promoter accessibility and transcription of *MLH1*. Analysis of the SYSCOL cohort strengthened the findings in the cell lines (Supplementary Fig. 3a) and identified a new gene target *DCLK3* in the MSS patients (Fig. 2e). Furthermore, CRISPR-CAS9 facilitated to generate fully comparable isogenic lines carrying G- or A-point mutation at rs1800734 locus. Therefore, mild changes in chromatin interaction, accessibility and consequently gene expression can be monitored by different genomic techniques. It has been shown that some functional SNPs in enhancer regions result in subtle changes in expression of their target genes, for example, the G-allele of rs356168 increased *SNCA* expression by 1.06 times in neurons and 1.18 times in neuron precursors^38^. Hence, accurate genome editing is required to distinguish these subtle changes. Using this model system, an increased *DCLK3* transcription was detected in the A/A homozygous line (Fig. 3c), which is due to increased chromatin interaction between the two locus and consequently elevated chromatin accessibility in the DCLK3 region (Fig. 3d,e,f). These data confirmed that rs1800734, even though locates in the promoter region of *MLH1*, serves as a distal enhancer for the *DCLK3* gene. Notably, our findings are based on the genetic background of MSS tumours, which is likely to be responsible for the contradiction between our data and some of the literature.

Moreover, DCLK3 has been shown to be associated with EMT process in this study (Fig. 4a). Although the full molecular mechanism of DCLK3 in regulating EMT has not been characterized, this protein has been shown to directly interact with CDK5 (ref. 39) and the latter promotes breast cancer metastasis through regulating transforming growth factor-β1-induced EMT^40^. Alternatively, CDK5 also prevents phosphorylation and degradation of a EMT regulator CALD1 (ref. 41) and hence promotes the EMT process^42^. Furthermore, DCLK3 may perform a similar function as another doublecortin-like kinase DCLK1, as it possesses the similar protein kinase domain and one of the two doublecortin domains^43^ as DCLK1. In intestinal tumours, DCLK1 often co-expresses with LGR5 at crypt base and DCLK1^+^LGR5^+^ stem cells are able to continuously produce tumour progeny under the APC^+/−^ mice^32^. A further study showed that DCLK1+ cells are long-lived and quiescent population, which is only activated and display carcinogenesis properties on oncogenic mutation and tissue injury^44^.

In summary, we conclude that ETS family TFs preferentially bind to the A-allele of rs1800734 and increase chromatin interaction between the rs1800734 locus and the *DCLK3* region. This enhanced chromatin interaction in turn increases the expression of *DCLK3*. Consequently, the risk of tumour metastasis is increased due to increased EMT feature of cancer cells (Fig. 4c). In addition, our study systematically identified changes in TF binding at regulatory CRC risk loci, which provide candidates for functional follow-up.

## Methods

### Cell culture and extraction of nuclear soluble fraction

Human CRC cell lines were cultured in DMEM or RPMI medium supplemented with 10% fetal bovine, 100 U ml^−1^ penicillin and 100 μg ml^−1^ streptomycin. LoVo and SNU175 cell lines were purchased from American Type Culture Collection and Korean Cell Line Bank (KCLB), respectively. COLO320 cell line was a generous gift of Dr Riccardo Fodde (Erasmus Medical Center Rotterdam, The Netherlands), which is originally from American Type Culture Collection. The authenticity of the cell lines were confirmed using microsatellite short tandem repeat (STR) assay by the suppliers of the cell lines. Mycoplasma infection was routinely tested in-house, to ensure that all the cell lines used for this study were free of mycoplasma contamination. Nuclear soluble fraction of LoVo, SNU175 and COLO320 was performed using a published protocol^45^. Protein concentration of the obtained nuclear extract was quantified using Branford assay. Each of the 3 mg extract (12 individual DNA pulldowns) was aliquoted, snap-frozen in liquid nitrogen and stored at −80 °C.

### High-throughput DNA pulldown

High-throughput DNA pull-down experiments was performed on 96-well filter plate format using our published method^46^ with minor modifications. To synthesize biotinylated double-stranded DNA (dsDNA) oligo, we attached a non-genomic 15 bp sequence at the 3′-end of the anti-sense strands. Subsequently, a reverse complement biotinylated primer was used to extend single-stranded DNA templates into dsDNA oligos. For each synthesis, 150 pmol of the biotinylated primer and 200 pmol template were subjected to a PCR reaction using Herculase II Fusion Enzyme kit under the following conditions: 95 °C for 3 min; thermocycling (*n*=20) at 95 °C for 1 min, 45 °C for 1 min and 72 °C for 1 min; 72 °C for 3 min; infinite hold at 12 °C. Briefly, high-throughput DNA pulldown was performed using a Multiscreen filter plate with 1.2 μm pores (Millipore, MSBVN1210). The biotinylated dsDNA oligos were immobilized on 20 μl of high-performance streptavidin sepharose (GE Healthcare, 17511301). Two-hundred and fifty micrograms of nuclear extracts and 15 μg of competitors (5 μg of poly(deoxyinosinic-deoxycytidylic) acid sodium salt (Sigma-Aldrich, P4929), poly(deoxyadenylic-thymidylic) acid sodium salt (Sigma-Aldrich, P0883) and Bakers yeast RNA (Sigma-Aldrich, R6750)) were added and incubated with immobilized oligos for 1.5 h at 4 °C on a plate shaker for each of the pull-down experiments. The components of the competitors were sonicated into ∼300 bp fragments before use. The proteins unbound to DNA oligos were washed off using different washing buffers and the bound TFs were on-bead digested overnight using trypsin/lysC. The pull-down duplicates underwent dimethyl label swapping and measured by nanoscale liquid chromatography tandem mass spectrometry (LC–MS/MS) in a 2 h gradient.

### Nuclear extract proteome

Deep proteome profile was generated from the nuclear extracts used for the pull-down experiments. An absolute quantification strategy was taken following a published method^46^. In brief, 3.3 μg of universal protein standard 2 (Sigma-Aldrich) and 10 μg of the nuclear extracts were mixed and subjected to a filter-aided sample preparation digestion. In parallel, 30 μg of the nuclear extracts were also digested using the filter-aided sample preparation protocol and subsequently separated into six fractions using strong anion exchange. The universal protein standard 2 sample and the fractions was purified on C18 stage tips and profiled by nanoscale LC–MS/MS in a 4 h gradient.

### DNase I sequencing

DNase I library of the LoVo cell line was constructed following a reported protocol with some minor modifications^47^. In short, 5 × 10^6^ nuclei were isolated using a buffer (15 mM Tris-HCl pH 8.0, 15 mM NaCl, 60 mM KCl, 1 mM EDTA pH 8.0, 0.5 mM EGTA pH 8.0 and 0.5 mM Spermidine) supplemented with 0.05% IGEPAL CA-630 detergent. Subsequently, the isolated nuclei were digested with 80 U DNase I (Sigma-Aldrich, D4527) for 3 min and the digestion was quenched by a stop buffer (50 mM Tris-HCl pH 8.0, 100 mM NaCl, 0.1% SDS, 100 mM EDTA pH 8.0, 1 mM Spermidine and 0.3 mM Spermine). A 9% Sucrose gradient was applied to fractionate the samples for 24 h at 25,000 r.p.m. at 16 °C and the fractions with <1 kb fragments were further purified and prepared according to the Illumina library preparation protocol.

DNase I library of other cell lines were prepared using a published protocol^18^ as described below. The cell lines were harvested under the confluence of 60% and washed with PBS. Nuclei were isolated with RSB lysis buffer (10 mM Tris-Cl pH 7.4, 10 mM NaCl, 3 mM MgCl_2_, 0.1% IGEPAL CA-630) at 4 °C for 10 min. Then the nuclei were treated with 0.12 unit of DNase I (Roche) in the provided buffer at 37 °C for 15 min before being quenched by 50 mM of EDTA. Following RNase A (Sigma) treatment at 37 °C for 15 min, proteinase K (NEB) was added for an additional hour at 56 °C. DNA was extracted using phenol:chloroform:isoamylalcohol. Agarose Gel (2%) electrophoresis was applied to separate the released fragments (∼100 bp) that were purified (Qiagen, MinElute Gel Extraction Kit), followed by Illumina TruSeq library preparation and Sequencing (HiSeq2000).

### ChIP-seq and ChIP–qPCR analysis

ChIP assays were performed following a standard protocol. Cell lines were cross-linked by a final concentration of 1% paraformaldehyde for 10 min and subsequently cross-linking reaction was quenched using 1.5 M glycine. The harvested cell lines were then lysed and sonicated to obtain ∼300 bp chromatin using Bioruptor Plus sonication device (Diagenode). The sonicated chromatin was pre-cleared by Protein A/G magetic beads (ThermoFisher Scientific, 88802) and then incubated together with antibody conjuncated beads overnight at 4 °C. Antibodies against H3K27ac (Diagenode, C15410196, 1 μg per ChIP assay), TFAP4 (Santa Cruz Biotechnology, sc-18593X, 6 μg per ChIP assay) and ELF1 (Santa Cruz Biotechnology, sc-631X, 4 μg per ChIP assay) were used in our ChIP experiments. Posterior to the incubation, captured chromatin was washed, eluted and de-crosslinked. Resulted DNA fragments were purified and prepared according to Illumina library preparation (H3K27ac ChIP) or KAPA Hyper Prep (TFAP4 and ELF1 ChIP) protocols before sequecning, or directly quantified using SYBR Green-based qPCR assays (Supplementary Table 3).

### ATAC-seq analysis

ATAC libraries of the SNU175 and COLO320 cell lines were prepared by a documented protocol^48^ with some modifications. In brief, nuclei were isolated using a lysis buffer consisting of 10 mM Tris-HCl pH 7.5, 10 mM NaCl, 3 mM MgCl_2_ and 0.1% IGEPAL CA-630 detergent and then tagmentated^49^ using 2 μl of Tn5 transposase and 12.5 ul 2 × TD buffer (Illumina, Nextera DNA Library Preparation Kit). The resulted DNA fragments underwent two sequential nine-cycle PCR amplification, and in between two PCR reactions the libraries were selected for <600 bp fragments using SPRI beads. The final PCR products were purified and quantified by KAPA Library Quantification Kits before sequencing.

### Targeted RNA-seq and reverse transcriptase–qPCR

Total RNA was isolated from the SNU175 and COLO320 cell lines using a TRIzol reagent (ThermoFisher Scientific, 15596018) based method. The yielded RNA was treated using DNase and then reversely transcribed into cDNA using random hexamers (ThermoFisher Scientific, SO142). The cDNA was amplified using targeted primers and followed by standard KAPA Hyper Prep protocols, or directly quantified using SYBR Green-based qPCR assays (Supplementary Table 3).

### CRISPR-CAS9-based SNP editing

CRISPR-CAS9 based SNP editing were performed according to a previously reported method^50^. Single guide RNAs (sgRNAs) were designed using an online tool (http://crispr.mit.edu/) and double-nicking strategy was taken to reduce undesirable off-target mutagenesis. The sgRNAs were then cloned into a U6-driven plasmid containing a green fluorescent protein (GFP) marker and the D10A mutant Cas9 nickase (Addgene, pSpCas9n(BB)-2A-GFP, Plasmid 48140). The plasmid was then transformed into the DH5α competent *Escherichia coli* strain and the products were purified for transfection. Subsequently, two sgRNAs (400 ng each) and a 199 bp single-stranded oligonucleotides (10 pmol single-stranded oligo donors/ssODNs (ssODNs), possessing G or A point mutations at the rs1800734 locus) were co-transfected into COLO320 cell lines following a standard lipofectamine LTX Reagent protocol (ThermoFisher Scientific, 15338100). FACS analysis was used to sort 192 GFP-positive cells per cell line (G or A point mutations) into 96-well plates in 36 h after the transfection. In parallel, a wild-type cell line was treated in the same manner by without using sgRNAs, and this mocked cell line was then sorted by FACS and served as the control. The desirable genotype at the rs1800734 was confirmed by Sanger sequencing. The oligos (sgRNAs and Single-Stranded Oligo Donors (ssODNs)) used in these experiments were listed in Supplementary Table 3.

### SNP genotyping

SNP genotyping was performed by a standard Sanger sequencing-based method. The regions containing mutations were PCR amplified into ∼500 bp fragments using specific primers (Supplementary Table 3) and the PCR products were purified using 1.5% agarose gel. A mixture of 10 ng purified PCR products and 6 pmol primers was used for Sanger sequencing and the data were analysed using CodonCode Aligner (V.5.0.2).

### 4C-seq analysis

The 4C experiments were carried out using a published protocol^51^ with some modifications. For each assay, 1 × 10^7^ cells were cross-linked and quenched as in ChIP assays. Nuclei were isolated in a 50 ml of lysis buffer (10 mM Tris-HCl pH 7.5, 10 mM NaCl, 0.2% IGEPAL CA-630 detergent and 1 × protease inhibitors). Subsequently, the nuclei were digested with 240 U NlaIII enzyme (New England BioLabs Inc., R0215L) followed by an overnight in-nuclei ligation with 4,000 U T4 ligase (New England BioLabs Inc., M0202M) at 16 °C. The ligated DNA was de-crosslinked, purified, digested with 90 U CviQI enzyme (New England BioLabs Inc., R0639S) and circularized by 5,000 U T4 ligase. The circularized DNA (16 × 300 ng) was amplified with bait-specific inverse primers (Supplementary Table 3), pooled and purified, followed by KAPA Hyper Prep protocols.

### Proteomics data processing

Recorded mass spectrometric (MS) files were analysed by MaxQuant software (version 1.3.5.7)^52^ using standard settings for dimethyl or label-free quantification analysis. All the files were searched against UniProtKB/Swiss-Prot human database (generated from version 06-2012). Batch effects of the pull-down data were removed using ‘Combat' algorithm^53^. In dimethyl analysis, some proteins were consistently quantified in the pulldowns of only one allele (Ref- or Alt-allele) in two replicate experiments and the protein ratios can therefore not be obtained from MaxQuant output. Therefore, we imputed the missing value in these pulldown using ‘Replace missing values from normal distribution' option in Perseus software (version 1.3.9.18)^54^, which allows to further calculate protein ratios and perform downstream analysis. TF interactors for each of the pulldown were identified using ‘Significance B' function in Perseus software.

### DNase I-seq ChIP-seq ATAC-seq data processing

Read mapping was performed using BWA-ALN (DNase I-seq and ChIP-seq) and BWA-MEM (ATAC-seq)^55^ against the hg19 reference human genome. PCR duplicates were removed for further data analysis. Peak calling was carried out by MASC2 (ref. 56) with default settings, except H3K27ac peaks were called using ‘broad' option. Peaks were called at a *q*-value cutoff of 0.01. Overlapping peaks were merged for each of the different experiments before further analysis. Integrative Genomics Viewer^57^ was used to detect bi-allelic differential binding and hypersensitivity.

### Targeted RNA-seq and RNA-seq data processing

Targeted RNA-seq data were mapped to the hg19 reference human genome using BWA-MEM^55^. Integrative Genomics Viewer^57^ was used to visualize the targeted RNA-seq data and detect bi-allelic differential expression. The SYSCOL RNA-seq cohort was mapped to the human reference genome sequence (GRCh37 autosomes+X+Y+M) using GEM mapper^58^. The reads with mapping quality <150 were excluded for further analysis. The genes were annotated using Ensemble 75. The reads of the genes were counted by ‘HTSeq' framework^59^ and normalized using ‘DESeq' package^60^.

### Calculate significance of the TF–SNP interactions

Importance of the TF–SNP interaction in our screen study was considered by combining DHS of the SNPs (read counts at the SNP position in 15 fetal large intestine tissues and 12 CRC cell lines) and the interaction strength (Log_2_(fold change) of a TF between Ref and Alt allele pulldown). The detailed *P*-value calculation was performed using *z*-test based on following steps: (1) calculate average DHS (average DNase I-seq reads) of all the 116 loci in the DNase I-seq data from 15 fetal large intestine tissues and 12 CRC cell lines, and then *z*-score transform the hypersensitivity data of these loci; (2) calculate absolute Log_2_(fold change) of every TF–SNP interaction between Ref and Alt alleles, and then *z*-score transform the absolute Log_2_(fold change) of all the interactions; (3) average the *z*-scores of DHS and absolute Log_2_(fold change), and convert the average *z*-scores into *P*-values based on normal statistical distribution.

### eQTL analysis

The eQTL analysis was performed as described in a previous publication^61^. Germline genotypes of these patients were genotyped on the Illumina 2.5M Exome v1.0 and imputed to 1000 genomes phase 3 release using IMPUTE2. For *cis*-eQTL analysis, we normalized gene quantification separately for healthy and tumour samples. Technical covariates were discovered using the PEER programme^62^ and 20 PEER factors were used in normalization. The *cis* region was defined as ±1 Mb from the transcription start site for each gene. The associations between genotypes and gene quantification were obtained using the FastQTL software^63^.

### 4C-seq data processing

A reduced genome was generated by extracting the sequences flanking the NlaIII restriction sites (30 bp on each strand from the NlaIII restriction sites to downstream) using the hg19 reference human genome in order to improve the mappability of our 4C-seq data. Subsequently, the mappability of the reduced sequences from each strand was evaluated and the uniquely mappable NlaIII restriction sites were kept for downstream analysis.

The reads from each library were parsed based on the bait-specific primer sequence and mapped to the reduced hg19 genome using BWA-ALN with the default parameters. A Bioconductor package ‘r3Cseq' (ref. 64) with 2.5 kb sliding window was used to determine significant interactions and calculate interaction difference.

### Pathway annotation and GSEA analysis

TFs binding differentially to the 116 loci were annotated to PANTHER pathways using PANTHER Classification System^65^.

GSEA analysis^66^ was applied to identify the gene sets correlated to DCLK3 expression. The search was performed against hallmark gene sets in Molecular Signatures Database (MSigDB, v5.1)^67^.

### Data availability

Raw and processed LC–MS/MS data and sequencing data are available at ProteomeXchange (http://www.ebi.ac.uk/pride/) and Gene Expression Omnibus (https://www.ncbi.nlm.nih.gov/geo/) under the accession numbers PXD004435 and GSE83968, respectively. The publically available data used in this study were downloaded from Gene Expression Omnibus (https://www.ncbi.nlm.nih.gov/geo/) using the following accession numbers: (1) H3K4me1 and H3K4me3 ChIP-seq: GSM1240111, GSM945304, GSM1208810, GSM1208811; (2) DNase I-seq: GSM736493, GSM736600, GSM736500, GSM736587, GSM665815, GSM665818, GSM665826, GSM701490, GSM701495, GSM701514, GSM701531, GSM774213, GSM774214, GSM774217, GSM774220, GSM774228, GSM774233, GSM817162, GSM817188; (3) TF ChIP-seq: GSM1010902, GSM1208683, GSM1208642, GSM1240820, GSM803354, GSM1010847, GSM1208763, GSM1010765, GSM1010790, GSM1010852, GSM1208598, GSM791411, GSM791412, GSM782123, GSM1122306, GSM722708, GSM1122302, GSM1122303. The detailed information of in-house generated and public data sets used in this study are listed in Supplementary Table 4. All other remaining data are available within the article and Supplementary Information files, or available from the authors upon request.

## Additional information

**How to cite this article:** Liu, N. Q. *et al*. The non-coding variant rs1800734 enhances DCLK3 expression through long-range interaction and promotes colorectal cancer progression. *Nat. Commun.* **8**, 14418 doi: 10.1038/ncomms14418 (2017).

**Publisher's note:** Springer Nature remains neutral with regard to jurisdictional claims in published maps and institutional affiliations.

## Supplementary Material

Supplementary Information Supplementary Figures, Supplementary Tables and Supplementary References

Supplementary Data 1 The SNP included in our PWAS study and the related information.

Supplementary Data 2 Summary of allele-specific protein binding events.

Supplementary Data 3 A list of transcription factors (TFs) and RNA binding proteins.
