# Supplementary material for: The non-coding variant rs1800734 enhances DCLK3 expression through long-range interaction and promotes colorectal cancer progression
Source: Nat Commun. 2017 Feb 14;8:14418. doi: 10.1038/ncomms14418 (PMC5316867; doi:10.1038/ncomms14418)
Supplement: Supplementary Information — Supplementary Figures, Supplementary Tables and Supplementary References [file ncomms14418-s1.pdf]

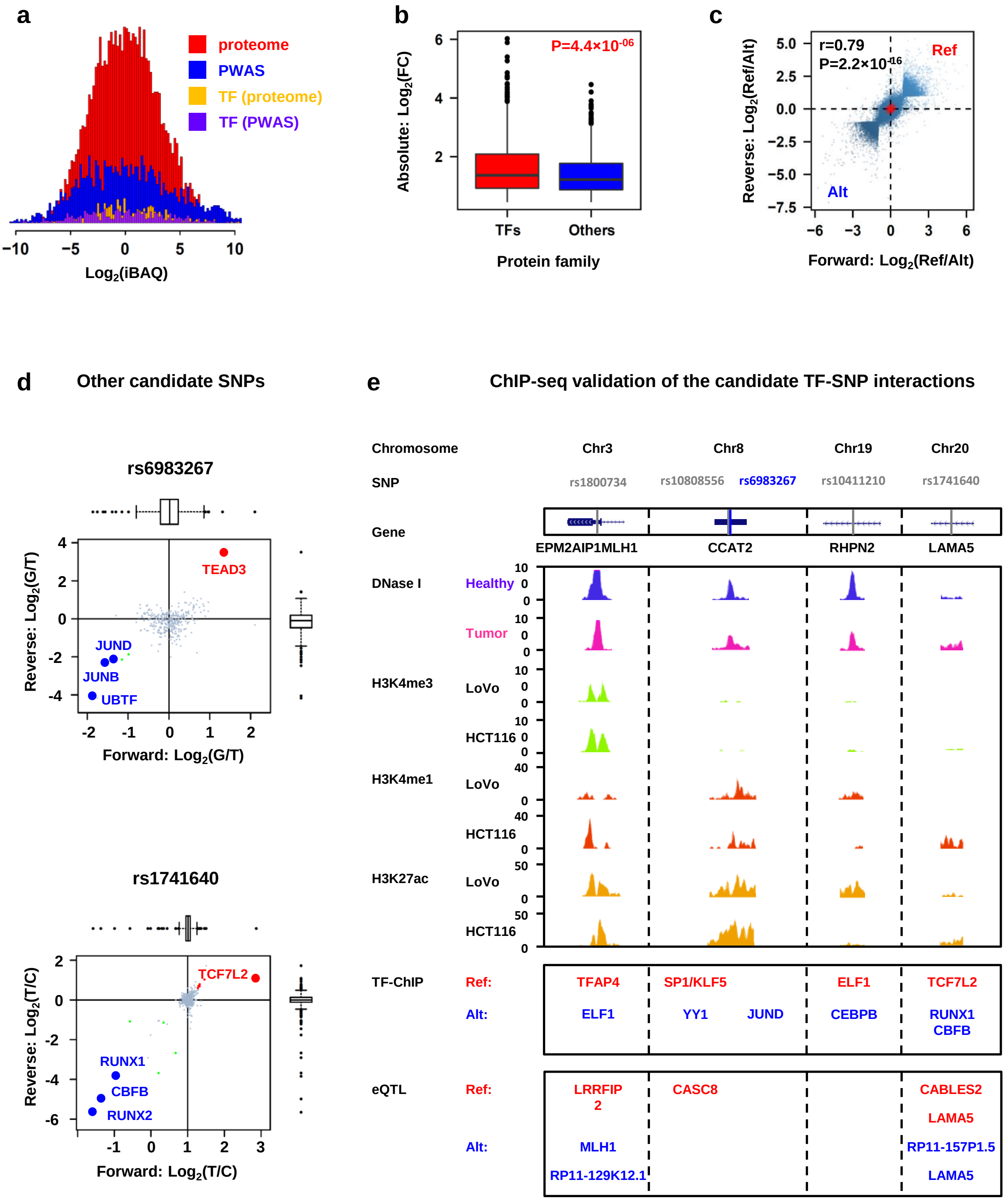

Supplementary Fig. 1 Summary of PWAS screen and ChIP-seq validation. (**a**) A histogram shows number and abundance distribution of identified proteins and TFs in our screen. We could clearly observe a TF enrichment in our PWAS data compared to nuclear extract proteome from the LoVo cell line; (**b**) Affinity changes in respect to the TFs and other proteins. In general, the TF interactors showed higher allele preference than other proteins (P-values: Mann-whitney U test); (**c**) Overlay of the 116 PWAS experiments. The red density dot indicates the central cloud of the PWAS screen (P-values: Spearman correlation test); (**d**) Other candidate TF-SNP interactions (n=2 pull-downs per SNP, red and blue dots: P-values<0.01, A/B significance test); (**e**) ChIP-seq validated TF-SNP interactions, and the chromatin environment (DNase hypersensitivity and histone markers) and eQTL information of these SNPs. The eQTL information was obtained from GTEx Portal website (http://www.gtexportal.org/home/).

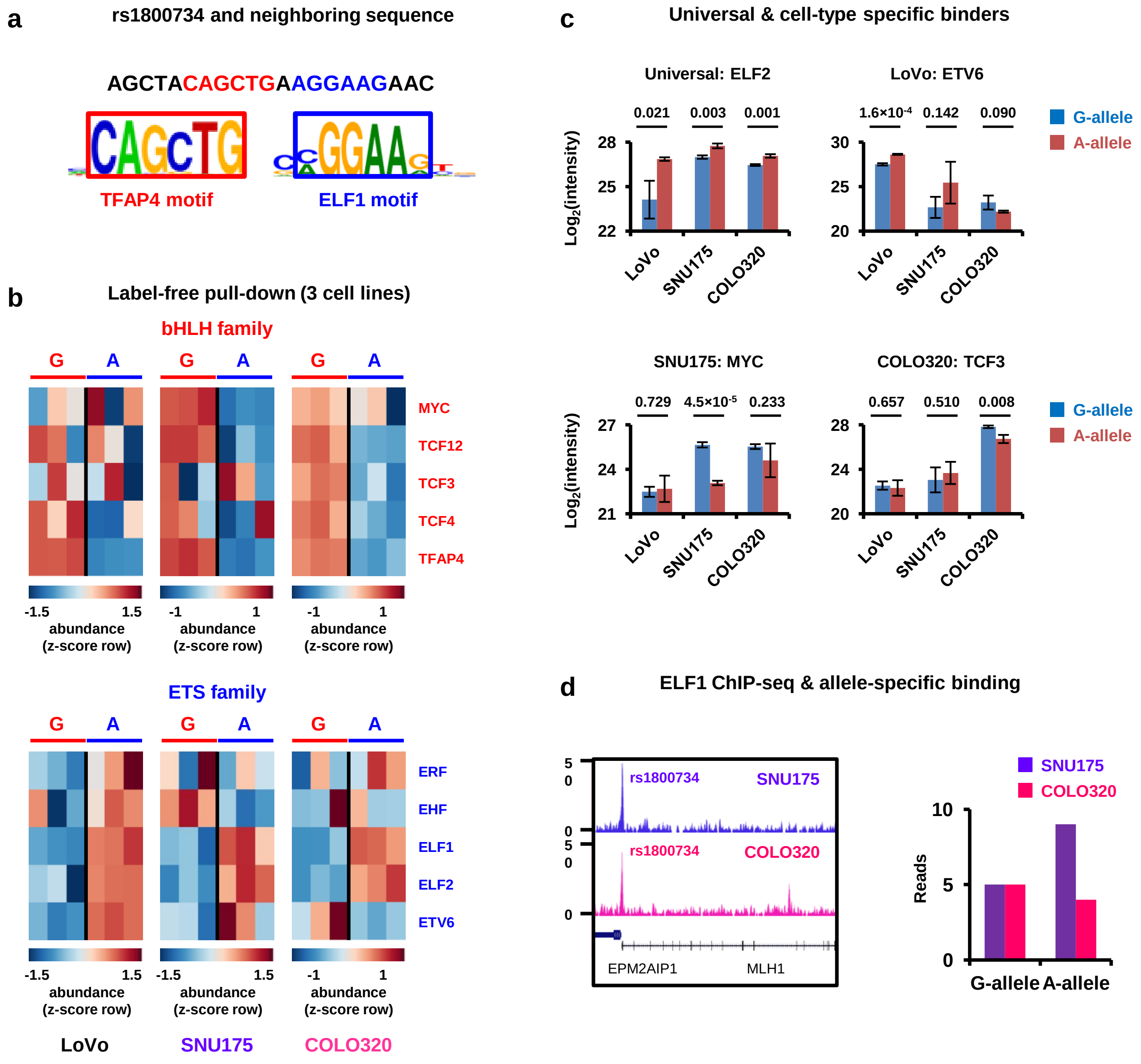

Supplementary Fig. 2 Identification of allele-specific TF binding at the rs1800734 loci. (**a**) Motif searching revealed bHLH and ETS motifs around the rs1800734 locus; (**b**) Significant allele-specific bHLH and ETS interactors of rs1800734 identified from the LoVo, SNU175, and COLO320 cell lines. Some of these interactors displayed cell-type specificity; (**c**) Examples of universal and cell-type specific interactors (Data are represented as mean and error bars indicate s.d., n=3 pull-downs per SNP, P-values: Student's t-test); (**d**) ELF1 ChIP-seq showed a stronger binding site at rs1800734. However, no clear allele-specific binding was observed, which might be caused by ETS family binding competition at rs1800734.

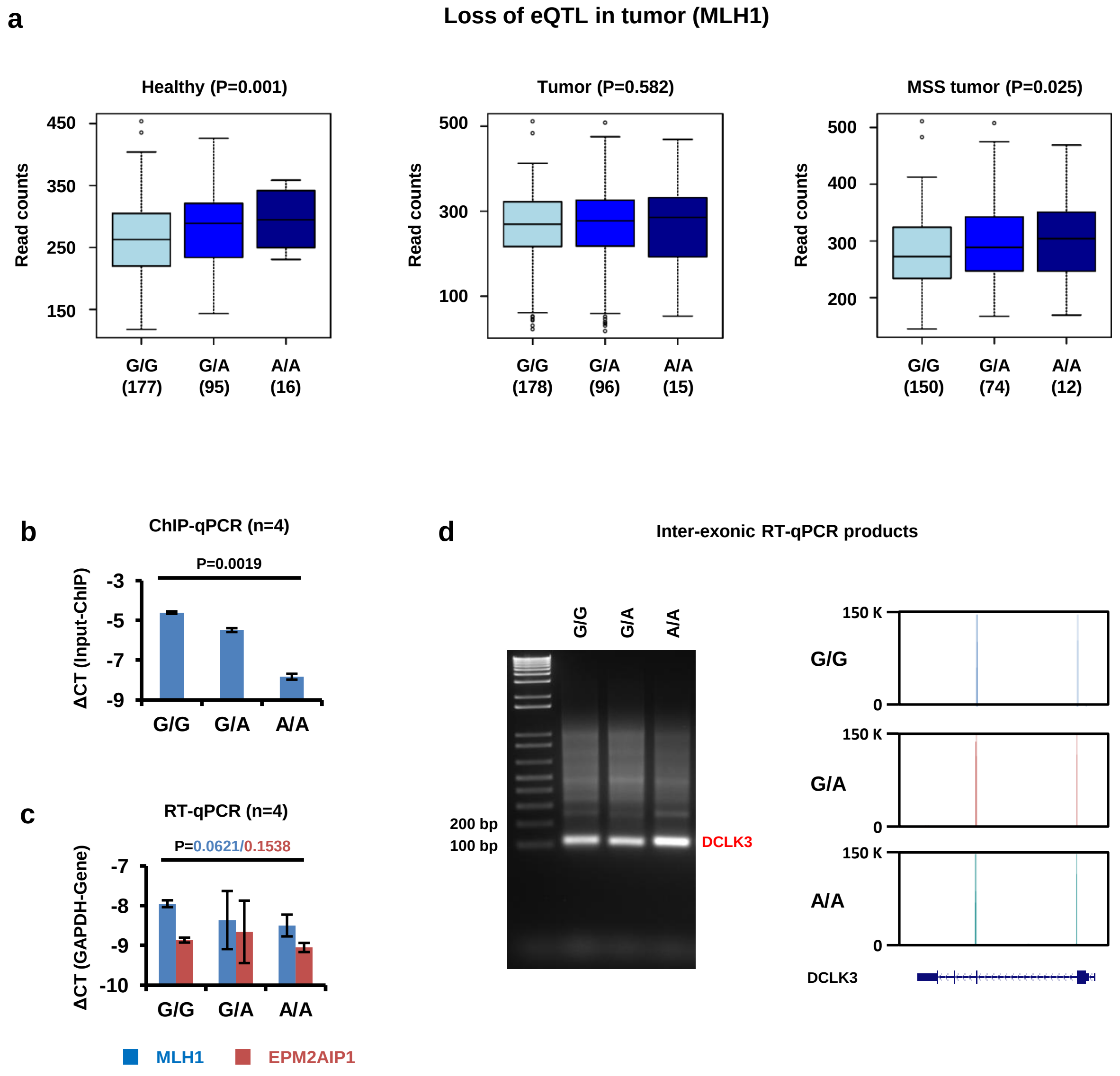

Supplementary Fig. 3 Potential gene targets of the rs1800734. (**a**) eQTL analysis showed a significant association between rs1800734 and the MLH1 expression in the healthy samples, which was lost in the tumor samples (P-values were calculated in FastQTL software based on Pearson correlation); (**b**) ChIP-qPCR analysis confirmed TFAP4 as a specific binder for the G-allele of rs1800734 (Data are represented as mean and error bars indicate s.d., n=4 ChIP assays per cell line, P-values: Student's t-test); (**c**) RT-qPCR analysis confirmed no expression change of MLH1 and EPM2AIP1 between the three isogenic cell lines (Data are represented as mean and error bars indicate s.d., n=4 biological replicates per cell line, P-values: Student's t-test); (**d**) RNA-seq of DCLK3 qPCR products confirmed the expression of this gene in these isogenic cell lines.

Supplementary Table 1. Summary of the top interactions selected based on our PWAS screen and DHS data

Interaction_ID	Gene_name	SNP_ID	Enrichment	Log2(FC)	DHS_type	DHS (reads)	Z-score[a]	P-value[b]
KLF4^698|rs10808556	KLF4	rs10808556	Ref	2.36222	healthy/tumor	6.185789547	2.460031709	0.013892474
KLF3^1147|rs10808556	KLF3	rs10808556	Ref	2.98073	healthy/tumor	6.185789547	2.83862736	0.004530803
SP1^557|rs10808556	SP1	rs10808556	Ref	4.189685	healthy/tumor	6.185789547	3.578639817	0.000345387
SP3^1251|rs10808556	SP3	rs10808556	Ref	3.46813	healthy/tumor	6.185789547	3.136969376	0.00170704
KLF16^1810|rs10808556	KLF16	rs10808556	Ref	3.882455	healthy/tumor	6.185789547	3.390581513	0.000697445
KLF13^2072|rs10808556	KLF13	rs10808556	Ref	3.369165	healthy/tumor	6.185789547	3.07639199	0.002095222
TFAP4^1243|rs1800734	TFAP4	rs1800734	Ref	3.80522	healthy/tumor	16.99049095	4.235575522	2.28E-05
PURB^1737|rs10411210	PURB	rs10411210	Ref	0.798427	healthy/tumor	10.64153382	1.981903097	0.047490087
ELF2^361|rs10411210	ELF2	rs10411210	Ref	0.91823	healthy/tumor	10.64153382	2.055235614	0.039856253
ELF1^366|rs10411210	ELF1	rs10411210	Ref	1.24287	healthy/tumor	10.64153382	2.253950739	0.024199267
TEAD1^576|rs9806137	TEAD1	rs9806137	Ref	4.49045	tumor	1.160340048	2.284858248	0.022321141
DEAF1^237|rs10411210	DEAF1	rs10411210	Ref	3.5698	healthy/tumor	10.64153382	3.678285961	0.000234807
LCOR^1720|rs10411210	LCOR	rs10411210	Alt	-1.337265	healthy/tumor	10.64153382	2.311730785	0.020792524
KDM1A^494|rs10808556	KDM1A	rs10808556	Alt	-2.30586	healthy/tumor	6.185789547	2.425533235	0.015285916
RCOR1^2021|rs10808556	RCOR1	rs10808556	Alt	-2.090205	healthy/tumor	6.185789547	2.293528829	0.021817578
RREB1^1661|rs10808556	RREB1	rs10808556	Alt	-2.11029	healthy/tumor	6.185789547	2.305823042	0.021120522
ZFP62^1598|rs1800734	ZFP62	rs1800734	Alt	-1.80505	healthy/tumor	16.99049095	3.011253097	0.002601719
HMGB3^316|rs16969681	HMGB3	rs16969681	Alt	-4.11176	no	2.291184409	2.653871892	0.007957403
UBTF^405|rs6983267	UBTF	rs6983267	Alt	-1.9452	healthy/tumor	6.090396229	2.191045433	0.028448505
ZNF581^1966|rs1944933	ZNF581	rs1944933	Alt	-6.0249	healthy	1.268020086	3.302477484	0.000958348
ELF1^366|rs1800734	ELF1	rs1800734	Alt	-0.9499125	healthy/tumor	16.99049095	2.48781558	0.012853036
RUNX1^1239|rs1741640	RUNX1	rs1741640	Alt	-3.91552	tumor	1.886824618	2.36227945	0.018162945
RUNX2^1320|rs1741640	RUNX2	rs1741640	Alt	-5.40292	tumor	1.886824618	3.27273065	0.001065139
CBFB^1322|rs1741640	CBFB	rs1741640	Alt	-4.860365	tumor	1.886824618	2.940627752	0.003275479
NFYA^974|rs77580392	NFYA	rs77580392	Alt	-5.8919	tumor	0.639417804	2.616458089	0.008884727
YY1^985|rs10808556	YY1	rs10808556	Alt	-3.297005	healthy/tumor	6.185789547	3.032222192	0.002427604
CEBPB^935|rs10411210	CEBPB	rs10411210	Alt	-1.319075	healthy/tumor	10.64153382	2.300596519	0.021414448

Note

(a) Z-scores were first calculated separately from Log2(FC) and DHS, and then the two Z-scores were averaged;

(b) P-values were derived from Z-scores by comparing to normal distribution.

Supplementary Table 2. Summary of the DCLK3 associated hallmark gene sets

Hallmark gene sets	Abbreviation	Type	Gene set size	NES[a]	P-value	FDR (q-value)
EPITHELIAL_MESENCHYMAL_TRANSITION	EMT	healthy	155	2.4964993	0	0
MYC_TARGETS_V2	MYC2	healthy	45	2.232153	0	0
E2F_TARGETS	E2F	healthy	169	2.1684825	0	0
MYC_TARGETS_V1	MYC1	healthy	154	2.0649652	0	0
G2M_CHECKPOINT	G2M	healthy	159	2.0314872	0	0
OXIDATIVE_PHOSPHORYLATION	OX_PHOS	healthy	150	-2.1709356	0	0
EPITHELIAL_MESENCHYMAL_TRANSITION	EMT	tumor	155	3.0950217	0	0
TNFA_SIGNALING_VIA_NFKB	TNFA	tumor	150	2.4517343	0	0
ANGIOGENESIS	ANGIO	tumor	22	2.337214	0	0
MYOGENESIS	MYO	tumor	122	2.0928953	0	0
INFLAMMATORY_RESPONSE	INFLAM	tumor	139	2.0710232	0	0
OXIDATIVE_PHOSPHORYLATION	OX_PHOS	tumor	150	-2.2223394	0	0
FATTY_ACID_METABOLISM	FA_META	tumor	117	-2.0197394	0	0

Note

(a) NES: Normalized enrichment score. In our study, we set the significance cutoff at 2 or -2.

Supplementary Table 3. Detailed information of the oligos used in this study

Oligo name	Oligo sequence (5'-3')	Application
**1. PCR/qPCR primers**		
rs1800734_NGS_Fw	TGGATGGCGTAAGCTACA	Generate amplicon for targeted sequencing
rs1800734_NGS_Rev	CAGTTCTCAATCATCTCTTTG	Generate amplicon for targeted sequencing
rs35149869_NGS_Fw	TACCAGGGAAAACCGCAAT	Generate amplicon for targeted sequencing
rs35149869_NGS_Rev	TGACCTTCACAGTCATTCTGC	Generate amplicon for targeted sequencing
rs1800734_GT_Fw	CTCTGCGCCAGATCACCT	rs1800734 genotyping amplicon
rs1800734_GT_Rev	CCCTCCGTACCAGTTCTCAA	rs1800734 genotyping amplicon
MLH1_promoter_ChIP_Fw	TGACTGGCATTCAAGCTGTC	TFAP4/ELF1 ChIP-qPCR
MLH1_promoter_ChIP_Rev	TTCAGCCAATCACCTCAGTG	TFAP4/ELF1 ChIP-qPCR
MLH1_RT-qPCR_Fw	CTTGTACCCCCCGGAGAAG	MLH1 mRNA expression
MLH1_RT-qPCR_Rev	TGCAACATCTCCCGGAGAAC	MLH1 mRNA expression
EPM2AIP1_RT-qPCR_Fw	ATGTGGATGACGCCCAAAAGA	EPM2AIP1 mRNA expression
EPM2AIP1_RT-qPCR_Rev	CACCACCAAATAACGCTGGGT	EPM2AIP1 mRNA expression
DCLK3_RT-qPCR_(1)_Fw	CCACTGACACTGAAGAGCATT	DCLK3 mRNA expression
DCLK3_RT-qPCR_(1)_Rev	CACAGCGGTGGTCTCCTTT	DCLK3 mRNA expression
DCLK3_RT-qPCR_(2)_Fw	GTCCACATGCACGACAAGAG	DCLK3 mRNA expression
DCLK3_RT-qPCR_(2)_Rev	TCCAAAATCAGCCAATTTCA	DCLK3 mRNA expression
GAPDH_RT-qPCR_Fw	CGCTCTCTGCTCCTCCTGTT	GAPDH mRNA expression
GAPDH_RT-qPCR_Rev	CCATGGTGTCTGAGCGATGT	GAPDH mRNA expression
**2. CRISPR-CAS9 sgRNAs and ssODNs**		
rs1800734_Guide_A_Cloning_Top	CACCGCATCCAGCCCCACCCTTCAG	sgRNA A for the double-nicking CRISPR CAS9
rs1800734_Guide_A_Cloning_Bottom	AAACCTGAAGGGTGGGGCTGGATGC	sgRNA A for the double-nicking CRISPR CAS9
rs1800734_Guide_B_Cloning_Top	CACCGCACGAGGCACTGAGGTGAT	sgRNA B for the double-nicking CRISPR CAS9
rs1800734_Guide_B_Cloning_Bottom	AAACATCACCTCAGTGCCTCGTGC	sgRNA B for the double-nicking CRISPR CAS9
rs1800734_G_ssODN	ACCCAGCAACCCACAGAGTTGAGAAATTTGACTGGCATTCAAGCT GTCCAATCAATAGCTGCCGCTGAAGGGTGGGGCTGGATGGCGTAA GCTACAGCTGAAGGAAGAACGTGAGCACGAGGCACTGAGGTGATT GGCTGAAGGCACTTCCGTTGAGCATCTAGACGTTTCCTTGGCTCTT CTGGCGCCAAAATGTCGT	ssODN possessing G base at the rs1800734
rs1800734_A_ssODN	ACCCAGCAACCCACAGAGTTGAGAAATTTGACTGGCATTCAAGCT GTCCAATCAATAGCTGCCGCTGAAGGGTGGGGCTGGATGGCGTAA GCTACAGCTAAAGGAAGAACGTGAGCACGAGGCACTGAGGTGATT	ssODN possessing A base at the rs1800734

	GGCTGAAGGCACTTCCGTTGAGCATCTAGACGTTTCCTTGGCTCTT CTGGCGCCAAAATGTCGT	
**3. 4C-seq bait-specific primers**		
rs1800734_CviQI_FW	GCGGCGGGGGAAGTTATC	Bait-specific primer at CviQI restriction site
rs1800734_NlaIII_FW	GGGACTTTGTATACCAGTGCATG	Bait-specific primer at NlaIII restriction site

Supplementary Table 4. Detailed information of in-house generated and public datasets used in this study

ID	Sample information	Cell line or tissues	Type	Database	Accession
**Proteomics datasets**					
1	PWAS data at the 116 CRC risk loci	LoVo	TF-SNP interaction (dimethyl labeling)	ProteomeXchange	PXD004435
2	Nuclear extract proteome	LoVo	Proteome (label free)	ProteomeXchange	PXD004435
3	rs1800734 PWAS data	LoVo, SNU175, COLO320	Proteome (label free)	ProteomeXchange	PXD004435
**Genomics datasets (in-house)**					
4	H3K27ac ChIP-seq	HCT-116, LoVo	Histone ChIP-seq	GEO	GSE83968
5	DNase I-seq (cell line)	LoVo, CaCO2, COLO205, GP5d,HT-29, HUTU80, RKO, SK-CO-1, SW480, SW1116, T-84	DNase I-seq	GEO	GSE83968
6	TFAP4 ChIP-seq	SNU175, COLO320	TF ChIP-seq	GEO	GSE83968
7	ELF1 ChIP-seq	SNU175, COLO320	TF ChIP-seq	GEO	GSE83968
8	Allele-specific hypersensitivity	SNU175, COLO320	ATAC-seq	GEO	GSE83968
9	Allele-specific expression	SNU175, COLO320	Targeted RNA-/DNA-seq	GEO	GSE83968
10	DCLK3 transcription	COLO320 rs1800734 isogenic lines	Targeted RNA-seq	GEO	GSE83968
11	Chromatin interaction	COLO320 rs1800734 isogenic lines	4C-seq	GEO	GSE83968
12	Chromatin accessibility of DCLK3	COLO320 rs1800734 isogenic lines	ATAC-seq	GEO	GSE83968
**Genomics datasets (public)**					
13	H3K4me1/H3K4me3 ChIP-seq	HCT-116, LoVo	Histone ChIP-seq	GEO	GSM1240111[1], GSM945304[2], GSM1208810, GSM1208811[3]
14	DNase I-seq (cell line)	HCT-116, CaCO2	DNase I-seq	GEO	GSM736493, GSM736600, GSM736500, GSM736587[2]
15	DNase I-seq (tissue)	Fetal large intestine	DNase I-seq	GEO	GSM665815, GSM665818, GSM665826, GSM701490, GSM701495, GSM701514, GSM701531, GSM774213, GSM774214, GSM774217, GSM774220, GSM774228, GSM774233, GSM817162, GSM817188[4]
16	SP1 ChIP-seq	HCT-116, LoVo	TF ChIP-seq	GEO	GSM1010902[5], GSM1208683[3]
17	KLF5 ChIP-seq	LoVo, GP5d	TF ChIP-seq	GEO	GSM1208642, GSM1240820[3]
18	YY1 ChIP-seq	HCT-116	TF ChIP-seq	GEO	GSM803354[5]
19	JUND ChIP-seq	HCT-116, LoVo	TF ChIP-seq	GEO	GSM1010847[5], GSM1208763[3]
20	ELF1 ChIP-seq	HCT-116, A549	TF ChIP-seq	GEO	GSM1010765, GSM1010790[5]
21	CEBPB ChIP-seq	HCT-116, LoVo, LS180	TF ChIP-seq	GEO	GSM1010852[5], GSM1208598[3], GSM791411, GSM791412[6]

22	TCF7L2 ChIP-seq	HCT-116	TF ChIP-seq	GEO	GSM782123[7]
23	RUNX1 ChIP-seq	ME-1, CD34+ blast cells	TF ChIP-seq	GEO	GSM1122306[8], GSM722708[9]
24	CBFB ChIP-seq	ME-1	TF ChIP-seq	GEO	GSM1122302, GSM1122303[8]
25	SYSCOL CRC RNA-seq	Paired normal and tumor samples from large intestine	RNA-seq	-	Available from the SYSCOL consortium upon request[10]

Supplementary references:

1. Hu, D. et al. The MLL3/MLL4 branches of the COMPASS family function as major histone H3K4 monomethylases at enhancers. Mol. Cell. Biol. 33, 4745–4754 (2013).

2. Thurman, R. E. et al. The accessible chromatin landscape of the human genome. Nature 489, 75–82 (2012).

3. Yan, J. et al. Transcription factor binding in human cells occurs in dense clusters formed around cohesin anchor sites. Cell 154, 801–813 (2013).

4. Maurano, M. T. et al. Systematic localization of common disease-associated variation in regulatory DNA. Science 337, 1190–1195 (2012).

5. Gertz, J. et al. Distinct properties of cell-type-specific and shared transcription factor binding sites. Mol. Cell 52, 25–36 (2013).

6. Meyer, M. B., Goetsch, P. D. & Pike, J. W. VDR/RXR and TCF4/β-catenin cistromes in colonic cells of colorectal tumor origin: impact on c-FOS and c-MYC gene expression. Mol. Endocrinol. 26, 37–51 (2012).

7. ENCODE Transcription Factor Binding Sites by ChIP-seq from Stanford/Yale/USC/Harvard

8. Mandoli, A. et al. CBFB-MYH11/RUNX1 together with a compendium of hematopoietic regulators, chromatin modifiers and basal transcription factors occupies self-renewal genes in inv(16) acute myeloid leukemia. Leukemia 28, 770–778 (2014).

9. Ptasinska, A. et al. Depletion of RUNX1/ETO in t(8;21) AML cells leads to genome-wide changes in chromatin structure and transcription factor binding. Leukemia 26, 1829–1841 (2012).

10. Bramsen JB et al., Improved colorectal cancer prognostication by molecular subtype-specific biomarkers, submitted.
